# Changes in the Facial Skeleton with Aging: Implications and Clinical Applications in Facial Rejuvenation

**DOI:** 10.1007/s00266-020-01785-0

**Published:** 2020-08-05

**Authors:** Bryan C. Mendelson, Chin Ho Wong

**Affiliations:** 1 Centre for Facial Plastic Surgery, 109 Mathoura Rd., Toorak, Victoria 3142 Australia; 2 W Aesthetic Plastic Surgery, 28/29, Mount Elizabeth Novena Specialist Center, 38 Irrawaddy Road, Singapore, 329563 Singapore

It is most gratifying that this paper has drawn so much interest from colleagues who look to the journal for direction in the way they care for their patients. This could not be considered a ‘hot’ topic, after all, being an occasional subject in Plastic surgery journals. Even then such papers tend to be focused on a small aspect of the topic, usually of technique. This level of interest may be that a Review article on this topic was timely in filling an overdue need. Moreover, it was most readable in providing a logical presentation of relevant information that summarizes the findings from many sources into a coherent analysis of the basics of skeletal aging of the face. Specifically, the changes from the aging process form the foundation for logical correction.

I give thanks to Dr Chin Ho Wong for this paper. It was conceived of and thoroughly researched by him, with a focus on raising surgeon’s awareness of the overlooked importance of correcting aging changes of the facial skeleton. This being fundamental, not only to obtaining a natural appearing rejuvenation of the aging face but maybe as important, in the prevention of many of the aging changes. During his fellowship time with me, Dr Wong was impressed that nearly every patient undergoing facial rejuvenation surgery had hydroxyapatite granules placed, usually on several locations on their facial skeleton. Given that facial rejuvenation has traditionally been considered surgery of the soft tissues he had not been expecting this focus on the skeleton. He had not seen this performed previously in his previous fellowship experience, nor had he heard this added dimension discussed at scientific meetings.

For those readers who have been positively influenced by the paper, and others reading for the first time, I am pleased to report that, even with the benefit of hindsight, there is nothing we would change in the paper. This is despite a further 10 years of experience, now numbering over 1500 cases. The message is clearly conveyed, aided significantly by the clarity of the illustrations, by Levent Efe, whose work and style has become widely appreciated. Readers will be interested to know that these illustrations, especially Figures 5 and 6, have been widely reproduced elsewhere, in many chapters and articles, and not just in plastic surgery publications. You may well ask then, why has there not been a wider use of hydroxyapatite onlay augmentation in facial rejuvenation. For this added perspective, please read on.

With the advent of a non surgical option, specifically, injectable commercial fillers out of a package, and the ready availability of cosmetic ‘filler’ practitioners, the simplicity, convenience and the usually good initial results, naturally attracts most potential candidates. However, it is important to appreciate that the filler results are not comparable to those provided by hydroxyapatite. The latter obtains a permanent correction with the one treatment, and properly done, the outcome is not the slightest surgical in appearance.

The key difference is the anatomical depth of the implanted volume. Hydroxyapatite is placed, not in the soft tissues as are fillers and fat, but subperiosteally, directly on the bone. As a consequence of the subperiosteal dissection, the overlying structures are lifted with the periosteum and then maintained in that position by the placement of the implant, which being solid, effectively acts as a biological spacer on the bone.

Significantly, when the periosteum is lifted, so are the attachments of the structures that take origin from the periosteum, the muscles and ligaments. On the mid-cheek, for example, the attachment of the zygomaticus muscles and the zygomatic ligaments are positioned outward as a result of the augmentation. Clearly, this does not occur with soft tissue injections, which are simply placed into the soft tissues superficial to the muscles and ligaments. The permanent elevation of these attachments is an important factor that contributes to a harmonious and inherently attractive look. The look is as if the patient had been born with this bony/soft tissue configuration. This is because the benefits extend beyond the immediate area augmented, as a consequence of the structures attached to the periosteum. Adjacent to the surgically enhanced convexity, there is a relative concavity. Together these ogee contour changes provide an element of tissue tightening, not just fullness at the site of the deeply positioned origin of the zygomaticus.

Subperiosteal placement also avoids the thickening of the superficial soft tissue, inherent with soft tissue injections, and which blunts the contour of the underlying bone. On the contrary, the skeletal contour is enhanced over the shape of the added bone (implant) that provides the overlying soft tissue with an improved tone along with some reduction in the puffiness that comes with laxity.

Lipofilling has a well-deserved popularity with surgeons. The subject is well taught by many renowned surgeon teachers in facial rejuvenation. The thinking has been that, as lipofilling is part of most quality rejuvenation surgery, while at it, why not simply add more fat volume to correct all soft tissue thinning, even that which has resulted from the bone shrinkage!

In reconstructive plastic surgery, it is a time-honored principle that ‘losses must be replaced in kind.’ This was one of the principles espoused a century ago by Sir Harold Gillies the father of Facial Reconstruction in wartime Britain. This understanding was nuanced by Val Lambros, one of the pioneering advocates of volumizing the face, who contrasted the essential difference between the two natural volumizing materials used by surgeons, ‘fat is a filler and bone is a definer.’ In other words, when bone is missing, replacement with a bone like replacement is not only logical, it is advantageous.

For the patient to have the benefit of hydroxyapatite augmentation, there is the disadvantage of it being performed in an operating room and requiring some form of anesthesia. There is negligible if any post-procedure bruising and swelling. For the surgeon, there is a learning curve, albeit rather small, as the subperiosteal procedures are technically simple and easily learned. Given the many benefits, for surgeons who work on the face, it is advantageous to learn the specific surgical techniques for the different areas of application. In practice, its use is limited only by the surgeon’s imagination. Historically, in contrast to lipofilling, hydroxyapatite usage has not been well taught at teaching Courses in plastic surgery conferences. To assist interested surgeons, a specific Teaching Course Demonstration of the various hydroxyapatite surgical techniques is available in DVD or streaming format in the QMP series by the senior author, who has waived all royalty entitlements.

What changes have there been in the 8 years since this paper was published?

A nearly completed study by my colleague, Dr. Richard Huggins, at the Victorian institute of Forensic Medicine in Melbourne promises to become the benchmark in quantifying the aging changes of the facial skeleton. The reason is that high-resolution, two-dimensional CT scans were used, as they provide better precision for measurement than 3D CT. Three hundred subjects, from birth through to 100 years, were analyzed with an equal gender distribution. The most evident changes are the marked resorption of the anterior maxilla immediately below the infraorbital rim and at the base of the nasal pyriform, most notable in the late 30′s through to 60′s group, from where the rate plateaus.

Soft tissue overlays on the skeleton show the intimate relationship of the tear trough to the infraorbital rim. In the pediatric population, the soft tissue trough is significantly above the rim, but with aging, the trough concavity descends to become more anatomically aligned with the rim.

There have been changes in the hydroxyapatite material used. The coral-derived hydroxyapatite for facial use (200-µm pore size), which has been the gold standard material used in most of the published studies, has not been available for over a year. From the beginning, the original research and application was focused on the requirements in oral surgery. Now, with the rapid growth of implant dentistry, demand for the fastest forming and strongest new bone that tolerates the forces of compression has resulted. This is because it provides for a shorter time interval before the second-stage implant placement. This has been the stimulus for the recent development of new synthetic hydroxyapatites. In practice, these specific requirements for inlay grafting exceed the less demanding material requirements for onlay grafting.

It is appropriate to conclude by acknowledging the vision of some of our pioneering plastic surgery thought leaders, whose influence has formed the foundation for this satisfying aspect of facial surgery. Robert Flowers, in response to recognizing the volume need on the maxilla, developed silicone tear trough implants. Joel Pessa brought to our attention the aging changes of the maxilla and the implications, while Steve Byrd taught us a predictable technique for Clinical Application.

